# Enhanced compressive strength behavior of modified cement paste reinforced with bio-based additives and dendritic fibro nano silica under controlled dispersion conditions

**DOI:** 10.1039/d5ra03597j

**Published:** 2025-08-14

**Authors:** Xiaojiao Zhang, Jiamin Chen, Shengnan Sun, Guangping Zhang, Amin Honarbakhsh

**Affiliations:** a School of Civil Engineering, Changchun University of Architecture and Civil Engineering ChangChun 130600 China xiaojiaolhpy307@sina.com; b Department of Civil Engineering, Islamic Azad University Neyshabur Branch Neyshabur Iran amin_honarbakhsh@yahoo.com

## Abstract

For many years, eco-friendly concrete composed of repurposed organic waste has been widely used due to its sustainability and reduced environmental impact. This cutting-edge method seeks to reduce environmental pollution while encouraging sustainable organic waste management practices. Despite these benefits, studies on the structural performance of lightweight concrete reinforced with spirulina algae-infused dendritic fibro nano silica (spirulina@DFNS) under various boundary conditions are still scarce. This research explored the impact behavior of lightweight concrete slabs when natural aggregate was fully substituted with spirulina@DFNS. To analyze structural performance, a series of low-velocity impact tests were carried out, and response surface analysis was applied to interpret crack development and resistance under different loading conditions. The results indicated that incorporating spirulina@DFNS enhanced the material's ability to absorb impact energy and resist crack propagation. However, its effect on limiting crack dimensions was minimal, and no significant gains in residual strength were observed. Despite these limitations, the study highlights both the challenges and the potential advantages of using spirulina@DFNS in fiber-reinforced concrete structures designed for impact resistance.

## Abstract 2

For many years, eco-friendly concrete composed of repurposed organic waste has been widely used due to its sustainability and reduced environmental impact.

## Graphical abstract

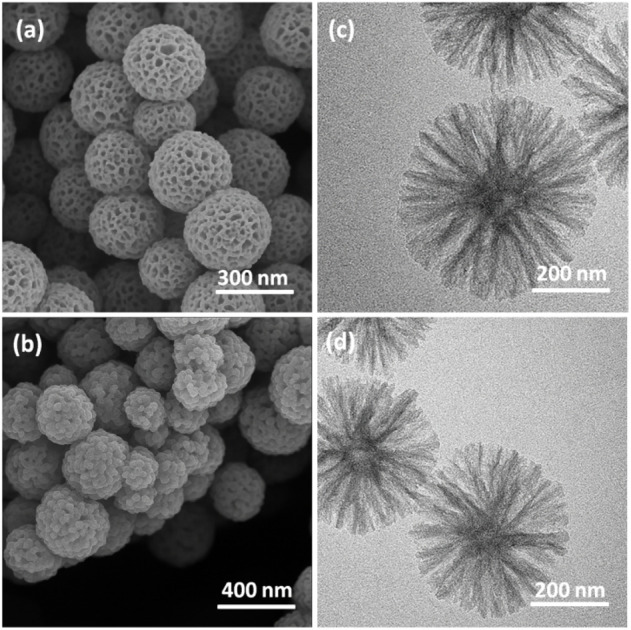

## Introduction

Many studies have concentrated on utilizing environmentally friendly products in construction by repurposing agricultural waste. The goal of this method is to decrease environmental pollution while fostering sustainable solutions for handling industrial waste. The unchecked proliferation of algae in both natural and man-made water systems produces large amounts of biomass, which must be harvested and managed to prevent environmental and aesthetic issues.^1^ The benefits and challenges of using algae waste as construction materials, covering factors such as economic viability, technical performance, sustainability, and environmental impact have been thoroughly discussed. The unchecked expansion of algae in both natural and engineered water environments results in the production of large amounts of biomass, which needs to be harvested and managed; otherwise, it will accumulate into unsightly and often smelly decaying masses.^2^ Therefore, using spirulina algae as lightweight aggregates in concrete provides several benefits, such as waste utilization and improved mechanical strength.^3^

Algae are widely found in nearly all types of aquatic ecosystems.^4^ Spirulina is a type of filamentous microalgae that is multicellular, photosynthetic, blue-green in color, and characterized by its spiral shape.^5^ Spirulina filaments are highly adaptable to their environment, offer excellent digestibility, contain 70% high-quality protein, and have a pleasant taste.^6^ Spirulina contains reactive functional groups, including hydroxyl and amino, which enhance its biocompatibility, pH sensitivity, toxicity, and capacity to interact with metals through these groups.^7^

Previous studies have highlighted the ecological benefits of incorporating natural fibers as coarse aggregates in concrete and have also explored the use of locally sourced solid waste materials as alternatives for producing lightweight concrete.^8–11^ The research indicated a 17% decrease in concrete density when natural aggregates were replaced with 75% natural fiber compounds.^12–15^ In particular, incorporating steel fibers at levels ranging from 0.30% to 1.1% into natural fiber compound concrete significantly improved both its flexural and compressive strength.^16^ Integrating various fiber types into lightweight concrete has been shown to improve key engineering characteristics, including resistance to fatigue, impact, and thermal shock, as well as enhanced fracture toughness and flexural strength.^17–19^ However, the overall performance of fiber-reinforced concrete is highly dependent on multiple factors, such as fiber content, material properties, and type, along with the water-to-cement ratio and aggregate selection.^20–27^

Under impact loading, tensile waves reflected from the impact site can lead to cracking on the opposite surface. Even heavily reinforced slabs, despite their high energy absorption capacity, remain vulnerable to failure due to punching shear. Additionally, impact events often cause localized damage at the point of contact, primarily due to compression failure in the immediate vicinity of the impact zone.^28,29^

This research is noteworthy because it replaces natural aggregate entirely with spirulina algae-reinforced dendritic fibro nano silica (spirulina@DFNS) and explores how different amounts of polypropylene fibers and boundary conditions influence the structural impact performance. These findings offer valuable insights into the potential of spirulina@DFNS concrete, especially for applications where enhanced impact resistance is essential.

## Experimental section

### Standard method used for synthesizing DFNS

Tetraethyl orthosilicate (3.6 g) was completely dissolved in a mixture containing 1-pentanol (2.4 mL) and cyclohexane (14 mL). In a separate container, urea (2.1 g) and cetylpyridinium bromide (0.7 g) were dissolved in water (42 mL). The resulting solution was subsequently introduced into the initial mixture. The combined solution was stirred continuously at room temperature for 33 minutes. The reactor was heated to 105 °C and maintained at that temperature for 3.8 hours.^7^

### Standard method used for synthesizing spirulina@DFNS

To ensure proper dispersion, 50 mg of DFNS were sonicated in 10 mL of 0.1 M acetic acid solution for 52 seconds. In a separate step, 125 mg of spirulina was dissolved in the same 0.1 M acetic acid solution, followed by stirring at 37 °C for 43 minutes. A syringe pump was employed to introduce 8.0 mL of TPP solution into the chitosan solution at a controlled rate of 2 mL min^−1^. After TPP addition was finalized, the mixture underwent mechanical stirring for a duration of 2.4 hours. The resulting product was isolated by filtration, rinsed with water until a neutral pH of 7 was achieved, and then vacuum-dried at 25 °C for 13 hours.

### Standard procedure for concrete preparation

A mixture of spirulina@DFNS and 115 g of ethanol was stirred for 1.2 h. Meanwhile, 314 g of ordinary Portland cement and 822 g of standard sand were introduced into the mixing equipment. The dry ingredients were mixed at a low speed for 4.5 minutes. Next, the suspension prepared earlier was gradually introduced into the mixer within 40 seconds, followed by the addition of 115 g more water. The mixture was initially stirred at low speed for 75 seconds, then at a higher speed for another 50 seconds. Next, the fresh mortar was cast into cube-shaped molds measuring 70 mm on each side. The molds were set on a vibration table and vibrated until all air bubbles had been removed from the surface of the mortar. The mortar specimens were removed from the molds after 28 hours of preliminary curing and placed in a regulated curing chamber for further treatment. Such an approach facilitates the successful incorporation of spirulina@DFNS within the concrete matrix. The mix design was prepared in accordance with ASTM C305 for mixing procedure and ASTM C109 for specimen casting and curing (Table 1).

**Table 1:** Mix proportions and physical characteristics of materials used

Component	Quantity (g)	Notes/specifications
Ordinary Portland cement	314	OPC 42.5 grade
Standard sand	822	ASTM C778; particle size: 0.15–2 mm
Water	115	Tap water
Ethanol	115	Analytical grade
Spirulina@DFNS	Variable (0–4%)	As % replacement of cementitious material (by weight)

## Results and discussion

**Fig. 1:**
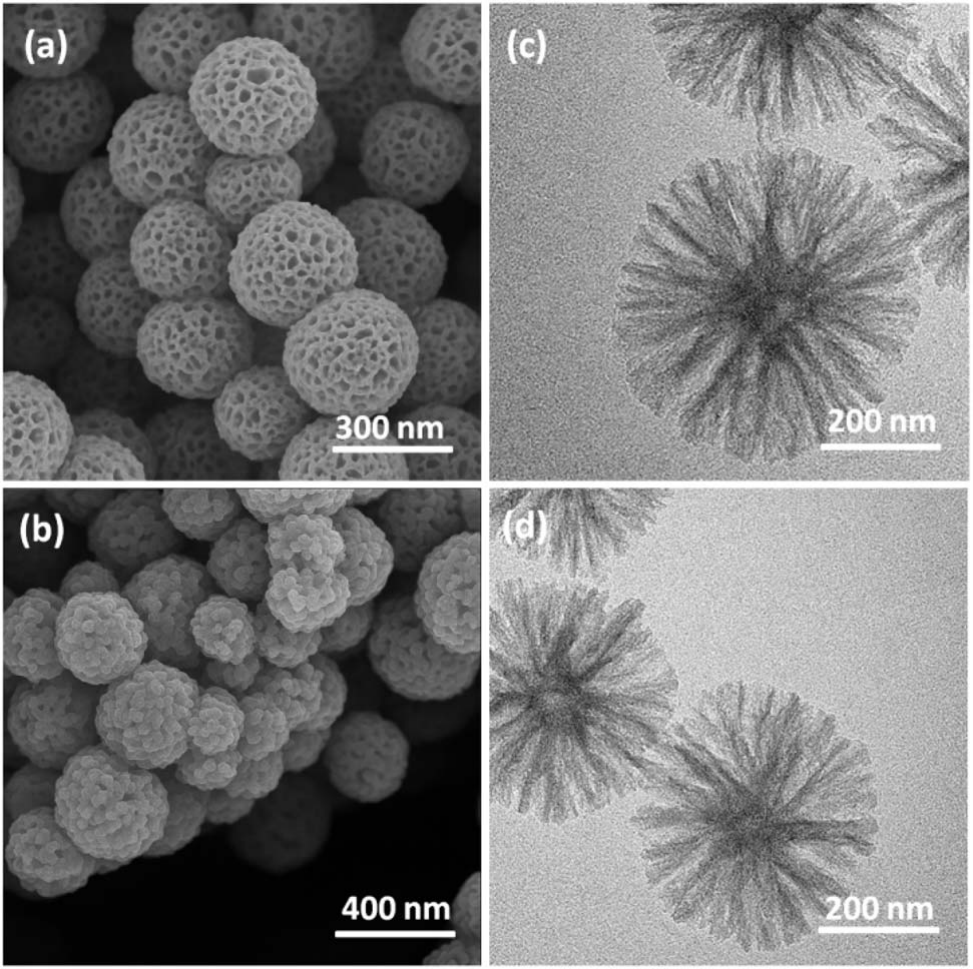
FESEM images of DFNS (a); spirulina@DFNS (b); TEM images of DFNS (c); spirulina@DFNS (d).

Fig. 1 presents the SEM and TEM images of DFNS and spirulina@DFNS. The DFNS consisted of uniform microspheres with a bicontinuous, concentric lamellar silica structure (Fig. 1a and c). This bicontinuous, concentric lamellar structure offers a high surface area and abundant silanol groups, which serve as nucleation sites for hydration products such as calcium silicate hydrate (C–S–H). Furthermore, the porous morphology enhances pozzolanic reactivity, allowing DFNS to interact with calcium hydroxide and improve the density and strength of the cement matrix. Additionally, the spirulina@DFNS exhibited a similar structure to that of DFNS, despite the incorporation of spirulina (Fig. 1b and d).

**Fig. 2:**
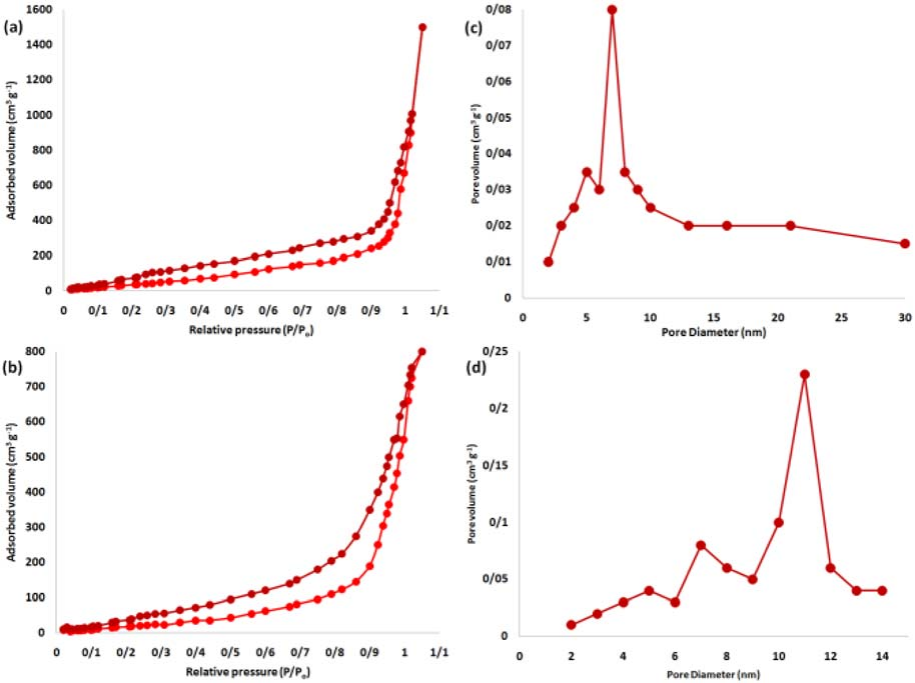
Adsorption–desorption isotherms of the DFNS (a), spirulina@DFNS (b) and BJH pore size distributions of the DFNS (c), spirulina@DFNS (d).

According to nitrogen physisorption analysis, the BET surface areas of DFNS and spirulina@DFNS were found to be 643 m^2^ g^−1^ and 386 m^2^ g^−1^, respectively. The reduction in surface area for spirulina@DFNS, compared to DFNS, can be attributed to the integration of spirulina into the DFNS structure. Although the BET surface area decreased significantly upon spirulina incorporation, this reduction can be attributed to the partial filling of DFNS pores with organic components. Nevertheless, the modified surface introduces reactive hydroxyl and amino groups from spirulina, which enhance chemical interaction with hydration products. Additionally, the preserved mesoporous structure maintains sufficient nucleation sites, allowing spirulina@DFNS to retain its pozzolanic contribution and positively influence matrix densification. Fig. 2 presents the nitrogen adsorption–desorption isotherms for the DFNS. DFNS showed a type IV isotherm accompanied by an H1-type hysteresis loop, indicating a mesoporous structure. BJH analysis of the desorption branch of the nitrogen isotherm revealed a narrow pore size distribution, peaking at 11 nm. The substantial mesopore volume and size of DFNS provide ample capacity to accommodate spirulina, which has a relatively large molecular size (Table 2).

**Table 2:** Structural parameters of DFNS, and spirulina@DFNS

Catalysts	*S*_BET_ (m^2^ g^−1^)	*V*_a_ (cm^3^ g^−1^)	*D*_BJH_ (nm)
DFNS	643	2.9	11
Spirulina@DFNS	386	1.7	7

**Fig. 3:**
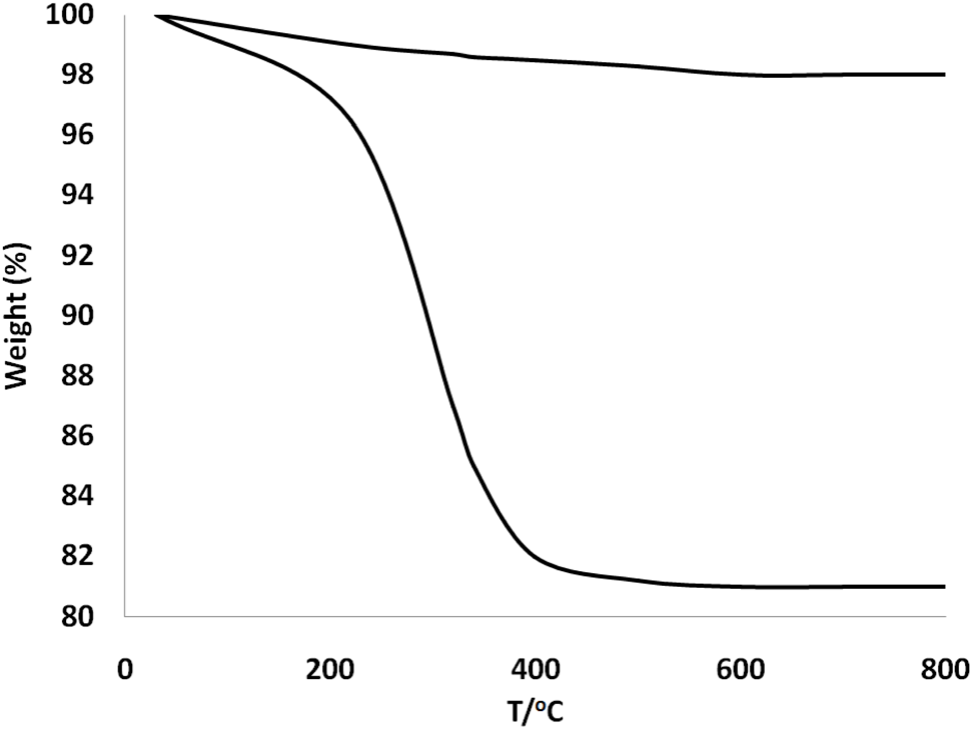
TGA diagram of DFNS, and spirulina@DFNS.

Thermogravimetric analysis (TGA) of DFNS and spirulina@DFNS was conducted from room temperature up to 850 °C to evaluate their thermal stability. The decrease in weight under 225 °C is attributed to the loss of solvents from the DFNS surface. The weight loss observed for spirulina@DFNS NPs was 18.9%. These results confirmed the excellent grafting efficiency of the organic compounds onto the DFNS (Fig. 3).

**Fig. 4:**
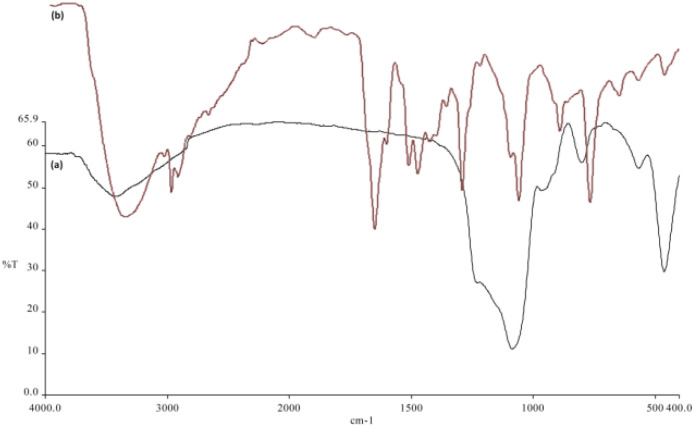
FTIR spectra of (a) DFNS, (b) spirulina@DFNS NPs.

FT-IR spectroscopy served to investigate the surface changes in the synthesized nanoparticles (Fig. 4). DFNS exhibited asymmetric and symmetric O–Si stretching vibrations at 802 and 1094 cm^−1^, respectively, as well as an O–H stretching vibration at 3312 cm^−1^ (Fig. 4a). Functional groups identified in spirulina@DFNS were revealed through FT-IR analysis. A frequency band between 3430 and 3250 cm^−1^ observed in the FT-IR spectrum is assigned to the H–O stretching vibration. Such a finding points to the existence of amino acids and carbohydrate compounds. The absorption peaks in the 3450–3250 cm^−1^ region correspond to H–N stretching, pointing to –NH_2_ groups associated with lipids and proteins. The absorption bands appearing at 2968 and 2884 cm^−1^ arise from the stretching vibrations of H–C bonds in aliphatic structures. The 1717 cm^−1^ absorption peak signifies C=O stretching, typically associated with amino acids and ester groups. The absorption peak at 1638 cm^−1^ is associated with N–H bending vibrations, suggesting the presence of a β-unsaturated ketone amide with a carbonyl group. A peak at 1397 cm^−1^ corresponds to CH_2_ bending vibrations. Absorption peaks at 1338 and 1272 cm^−1^ correspond to O–C stretching and O–H bending, indicative of alcohol groups (Fig. 4b).

**Fig. 5:**
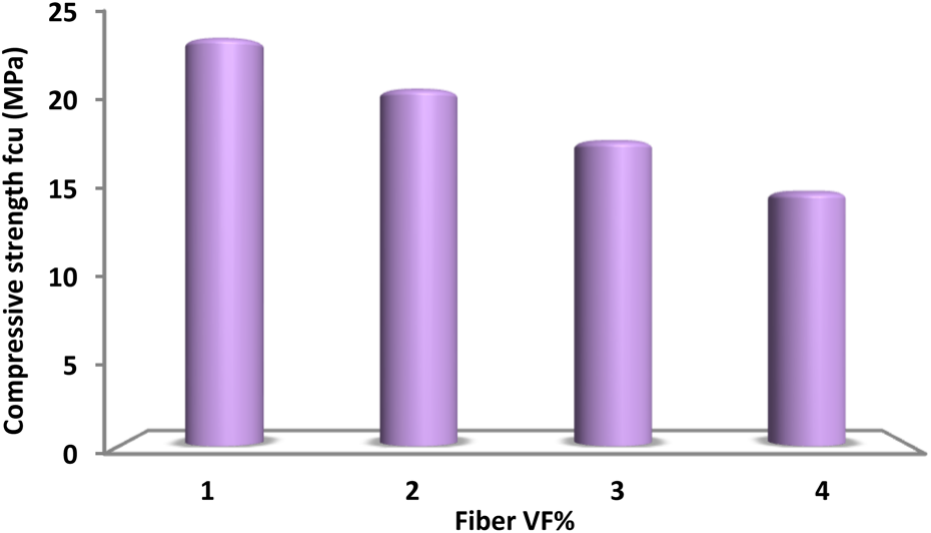
The 30 day compressive strength of concrete varies with different spirulina content.

Fig. 5 depicts the variations in compressive strength of lightweight concrete incorporating different amounts of spirulina fiber reinforcement. The complete substitution of natural aggregate with spirulina fibers results in a notable decrease in compressive strength, demonstrating the adverse effect of higher spirulina fiber content. Multiple elements influence this phenomenon. An increase in spirulina content leads to a larger overall surface area within the concrete mixture, necessitating a greater amount of binder along with an elevated water-to-cement ratio. Consequently, this modification may result in a reduction in compressive strength. One key issue is that the interaction between fibers hinders the movement of cement paste, creating localized stress points that ultimately lead to the crushing of spirulina. Moreover, too much fiber can cause clumping, which leads to the formation of voids and weakens the connection between the cement paste and the fibers. At elevated fiber dosages, reduced workability hinders the uniform dispersion and proper orientation of fibers within the concrete, ultimately diminishing compressive strength and lowering load transfer efficiency. Previous studies have also indicated a decline in compressive strength with the incorporation of spirulina.

**Fig. 6:**
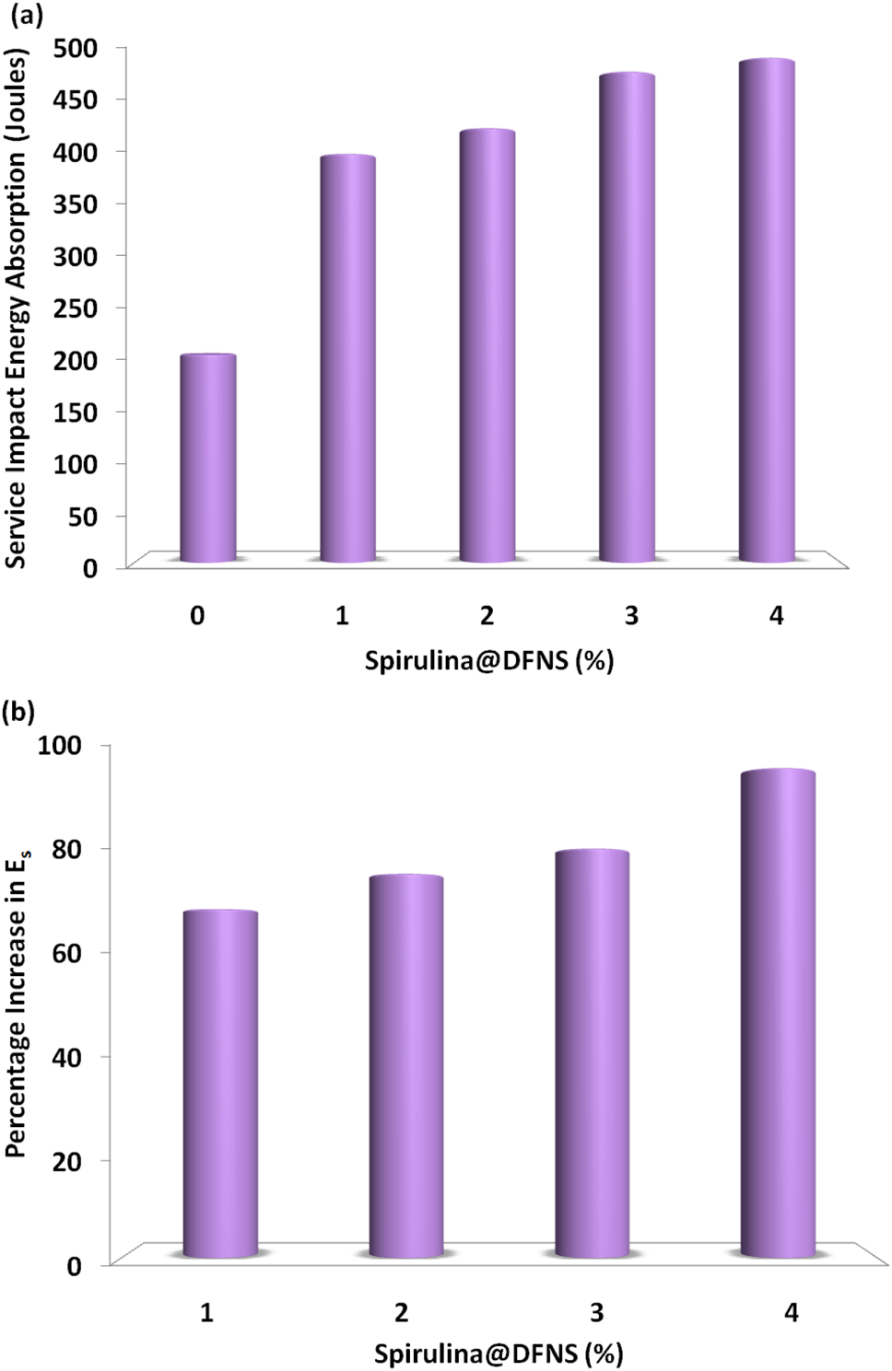
(a) Measured service impact energy absorption of spirulina@DFN concrete slab; (b) percentage increase in service impact energy.

As shown in Fig. 6a, there is a noticeable rise in service impact energy, which is closely linked to the amount of fiber content. A notable enhancement in service impact energy is seen as the fiber content rises from 1% to 4%, with the highest energy of 463.7 J achieved in the sample containing 4% polypropylene fiber. It is worth noting that although the service and ultimate impact energy improved consistently with increasing fiber content up to 4%, further increases beyond this dosage may negatively affect workability and uniform fiber dispersion. Excessive fiber content often leads to clumping, poor alignment, and increased porosity, which can diminish the mechanical integrity of the composite. Similar trends have been reported in prior studies involving natural and synthetic fiber-reinforced concretes.^16,25^ The results further demonstrate the strong influence of fiber content on impact energy, with a remarkable 97% increase at 4% fiber content compared to the control sample. Fig. 6b illustrates how the impact energy percentage increases with rising fiber content under different boundary conditions, with specimens having fewer restrictions showing a more significant enhancement.

**Fig. 7:**
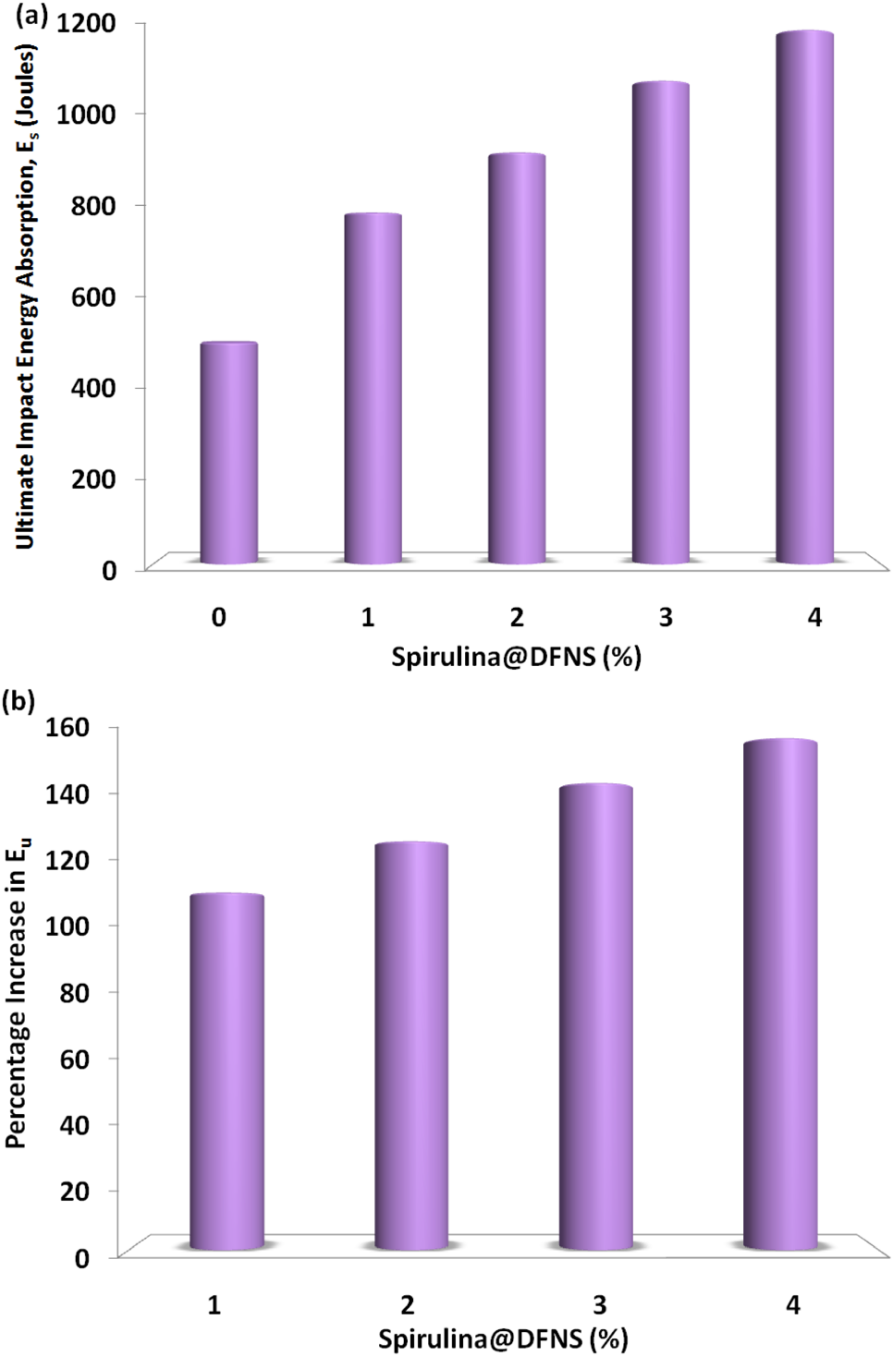
(a) Experimental maximum impact energy absorption of lightweight spirulina@DFNS concrete slab and (b) percentage growth in maximum impact energy.

Fig. 7a shows how different volumes of spirulina fiber influence the maximum impact energy of spirulina@DFNS concrete slabs. The trend for ultimate impact energy closely mirrors that of the service impact energy, with the highest recorded value of 1198 J found in the slab containing 4% fiber. Fig. 7b shows how impact energy changes with varying fiber content. A significant improvement in spirulina@DFNS concrete is evident at 1–4% fiber content. This improvement can be attributed to the crushing value and low impact of spirulina@DFNS, as well as the enhanced tensile strength of spirulina.^25^ Comparable findings have also been reported, emphasizing enhanced crack resistance and improved impact performance. Mo *et al.* also observed improved reduced crack width and impact resistance in natural fiber concrete when hybrid steel fibers were incorporated.

**Fig. 8:**
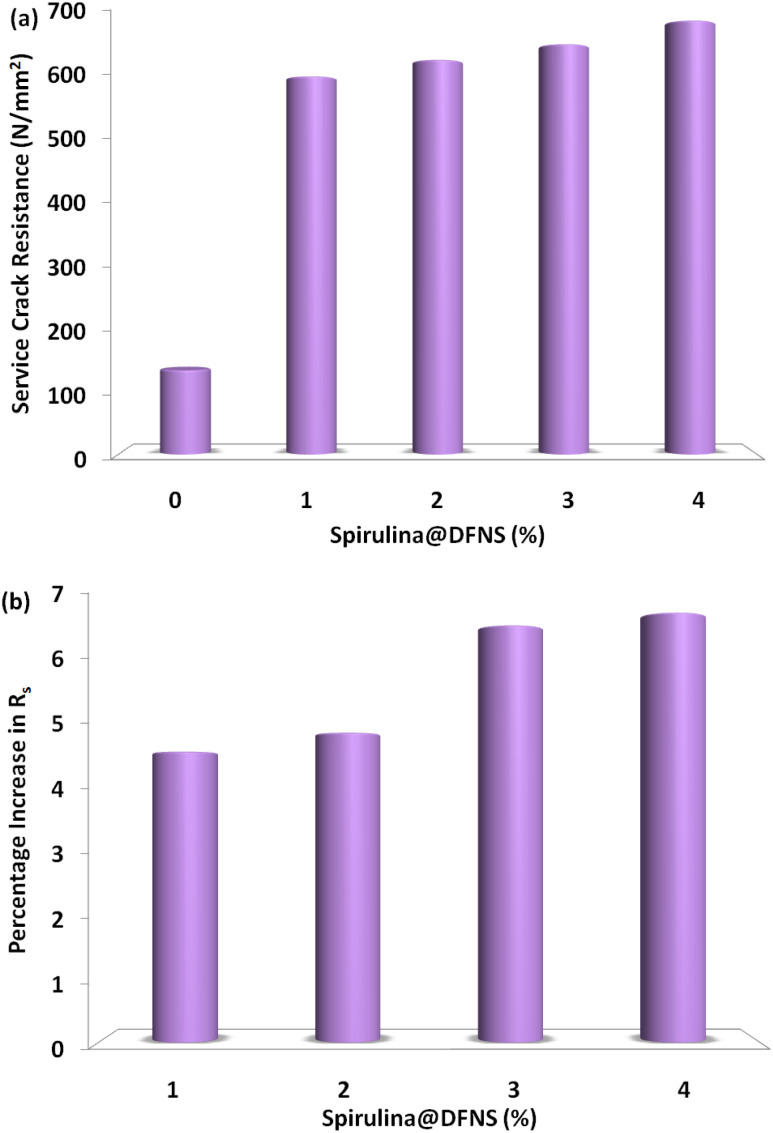
(a) Measured service crack resistance of lightweight-OPS concrete slab; and (b) percentage growth in service crack resistance.

Fig. 8a illustrates a steady enhancement in service crack resistance across all specimens as fiber content rises. This improvement becomes even more significant with greater amounts of spirulina@DFNS, achieving peak resistance levels. Likewise, an increase in the number of supports contributes to superior crack resistance. In Fig. 8b, a comparison between fiber-reinforced specimens and the control sample highlights a notable improvement. Among them, the specimen containing 4% fiber demonstrated the highest service crack resistance, reaching 680 J.

**Fig. 9:**
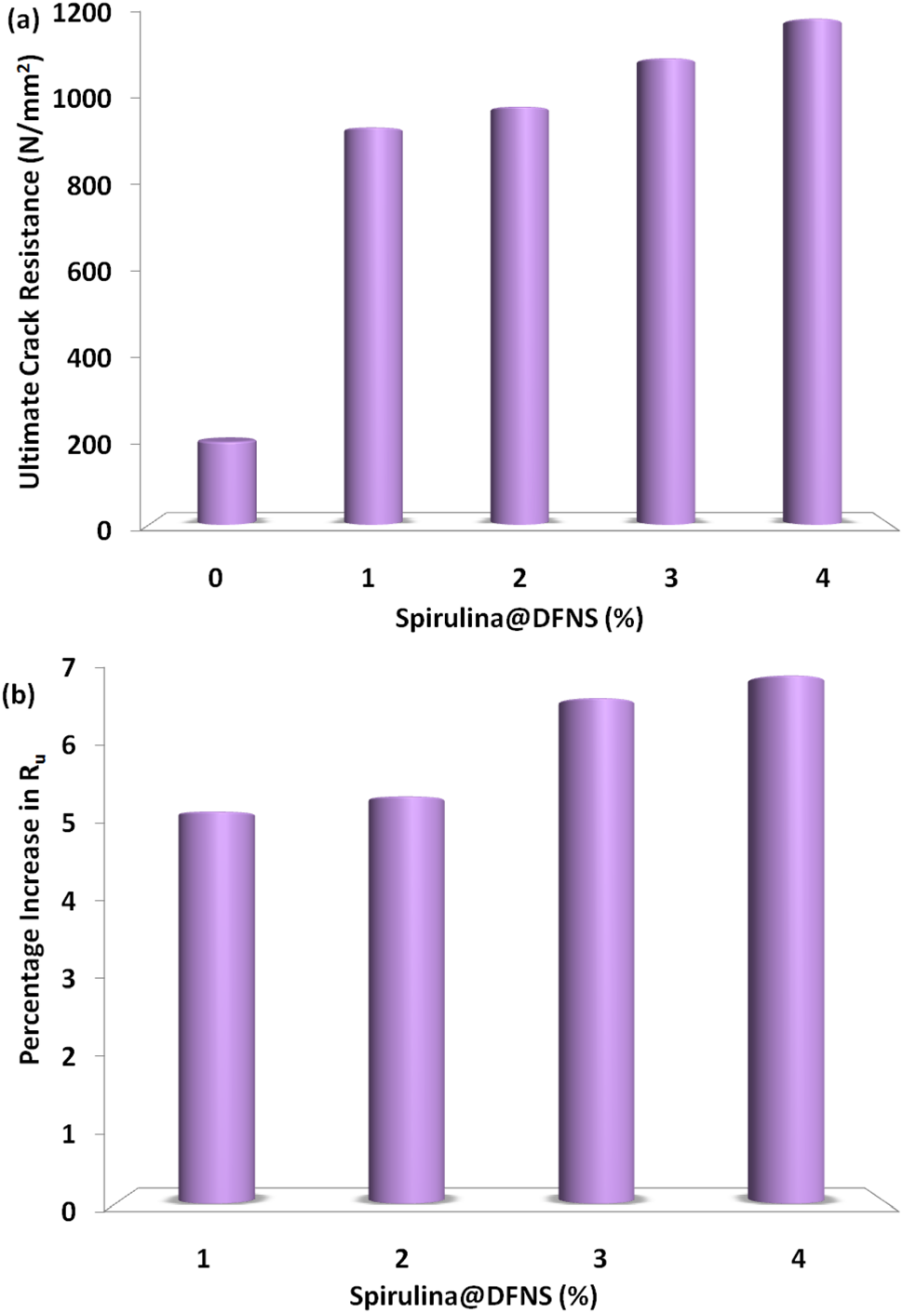
(a) Measured ultimate crack resistance of lightweight-OPS concrete slab; and (b) percentage growth in ultimate crack resistance.

Fig. 9a illustrates the fluctuation in ultimate crack resistance. As expected, the ultimate crack resistance values were consistently higher than the service crack resistance values, following a similar trend. The highest recorded value was 1198 N mm^−2^ in the specimen with 4% spirulina fiber. The increase is about sixfold compared to the control sample in that group, demonstrating the substantial impact of spirulina on the overall performance of spirulina@DFNS concrete. Fig. 9b presents the percentage variation in ultimate crack resistance for different support conditions across various fiber volume fractions. The variation in crack resistance at this stage is primarily due to spirulina@DFNS's ability to mitigate micro-cracks early on, combined with the spirulina mesh's role in bridging and controlling small cracks as failure progresses. Furthermore, the varying support conditions may have influenced how impact energy was distributed and how failure zones developed, leading to distinct performance outcomes.

**Fig. 10:**
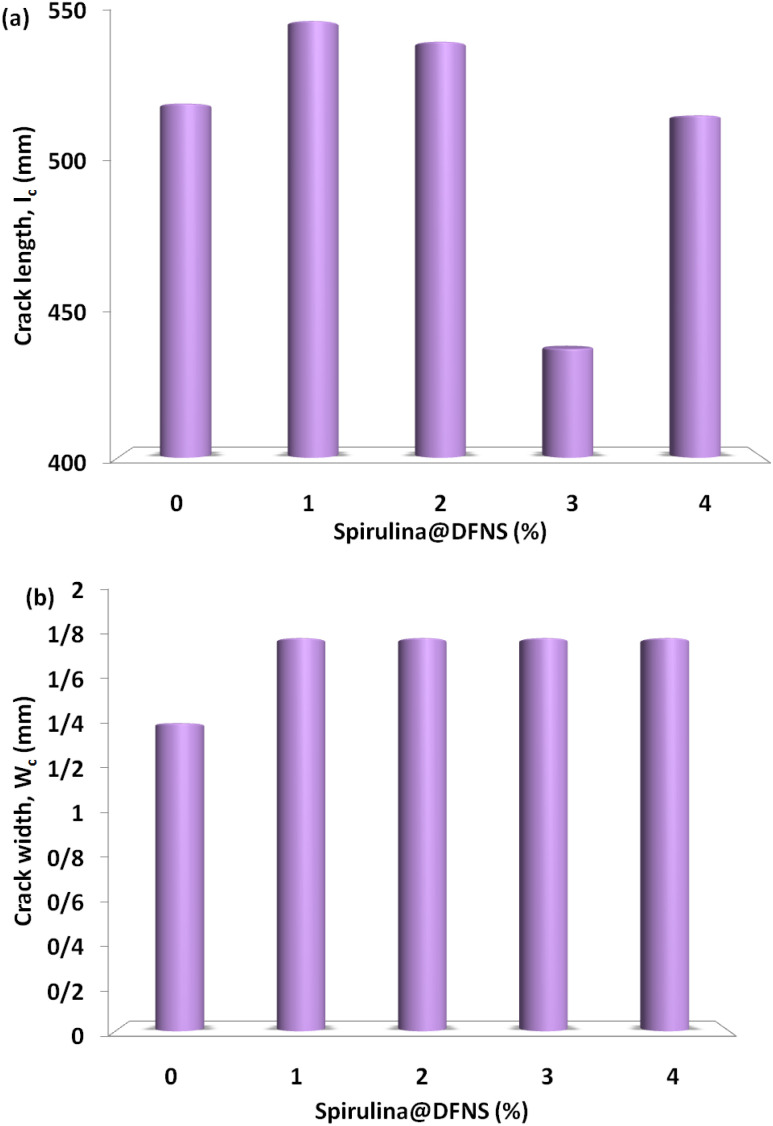
Effect of spirulina@DFNS boundary and content conditions on (a) crack length (b) crack width.

Fig. 10a shows that the cracks formed under impact loading in the slab specimens experience a 16% reduction in average length with the inclusion of up to 3% spirulina@DFNS. The reduction remains consistent, even with a 3% increase in spirulina@DFNS content in the simply supported slab. This indicates that incorporating spirulina in spirulina@DFNS has a modest impact on controlling both crack width and length. In contrast, Fig. 10b shows no change in crack width, with it remaining constant at 1.8 mm regardless of the fiber content.

**Fig. 11:**
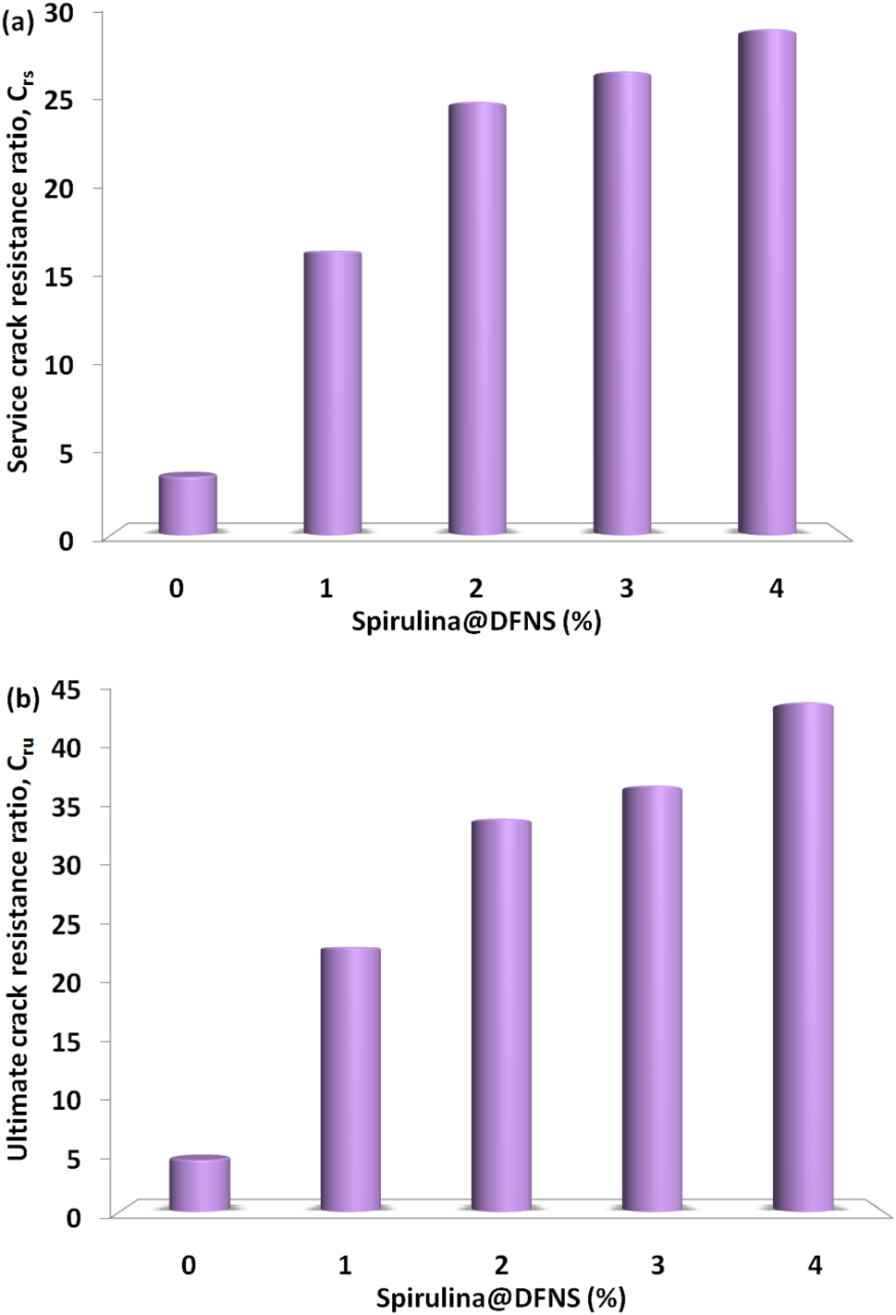
Comparison of ultimate and service crack resistance ratios of concrete across various boundary conditions (a) and fiber dosages (b).

Fig. 11a illustrates how the crack resistance ratio varies with the volume fraction of spirulina@DFNS under both service and ultimate failure conditions across different support configurations. This ratio represents the material's capacity to resist cracking relative to its compressive strength. Notably, an increase in fiber content and the number of supports leads to an improvement in both service and ultimate crack resistance ratios. This trend underscores the contribution of spirulina@DFNS to enhancing crack resistance in proportion to the material's compressive strength, as depicted in Fig. 11b.

**Fig. 12:**
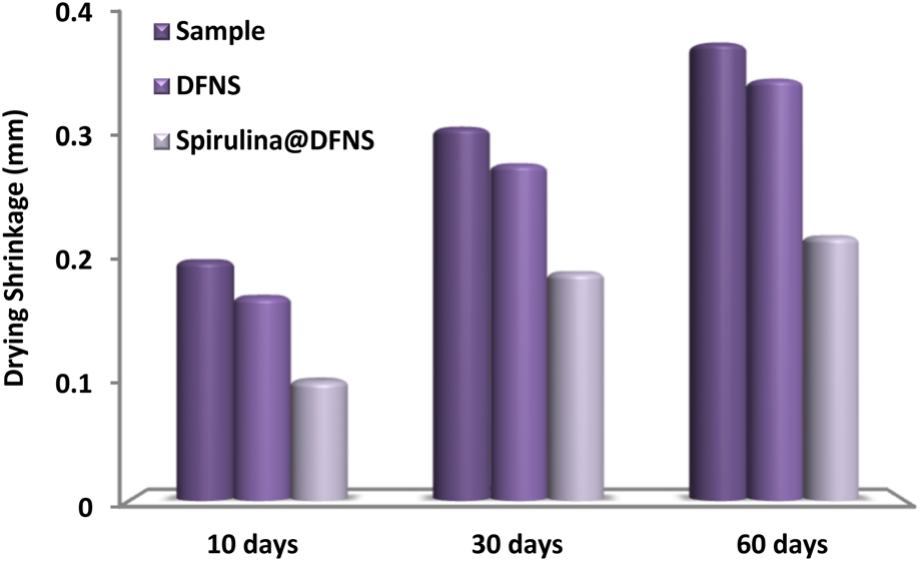
The effect of varying mass fractions of spirulina@DFNS and DFNS on concrete drying shrinkage.

Drying shrinkage in concrete refers to the volumetric reduction resulting from moisture loss, a key factor in crack formation. The cement paste incorporating spirulina@DFNS demonstrates viscoelastic properties, indicating that volume changes arise from both viscous and elastic mechanisms. Fig. 12 illustrates the results of the drying shrinkage test for concrete incorporating various weight ratios of spirulina@DFNS. All mixtures exhibit a progressive increase in shrinkage with time. Significantly, the control mixture undergoes the greatest moisture loss, leading to the highest degree of shrinkage. Incorporating spirulina@DFNS led to a noticeable reduction in concrete's drying shrinkage. This effect is attributed to spirulina@DFNS's ability to dissipate tensile energy as the concrete shrinks. The absorption process occurs at the interface between the spirulina@DFNS and the concrete matrix, transferring the energy into the surrounding material. This helps relieve excessive tensile stresses and reduces the potential for crack formation. When spirulina@DFNS is evenly dispersed in the cement paste, its high surface energy facilitates the nucleation of hydration products, promoting the hydration process and further limiting shrinkage.

**Fig. 13:**
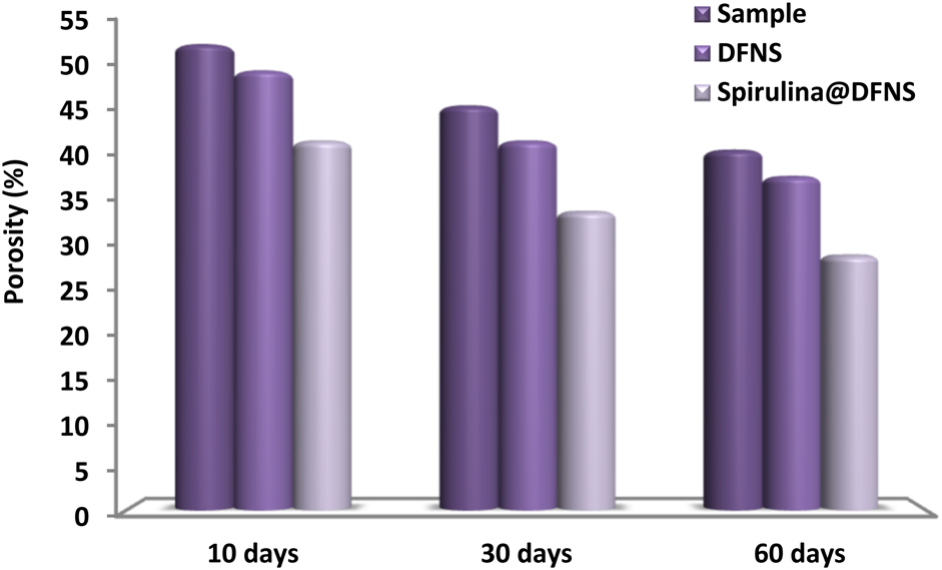
The effect of varying weight fractions of spirulina@DFNS and DFNS on the porosity of concrete.

Fig. 13 shows how varying nanoparticle weight ratios affect the porosity of concrete. The incorporation of nanoparticles significantly reduced porosity, achieving an approximate 12% decrease. By day 30, the porosity levels of the spirulina@DFNS and DFNS NP samples were recorded at 42% and 34%, respectively, in contrast to 46% in the control sample. This reduction is attributed to the enhanced hydration process facilitated by spirulina@DFNS, which improved the mixture's ability to fill voids. While the control sample contained large, interconnected pores, the addition of spirulina@DFNS refined the microstructure, minimizing both void formation and distribution. As a result, this improvement enhanced the adhesion between the cement matrix and filler, significantly boosting the concrete's overall strength.

**Fig. 14:**
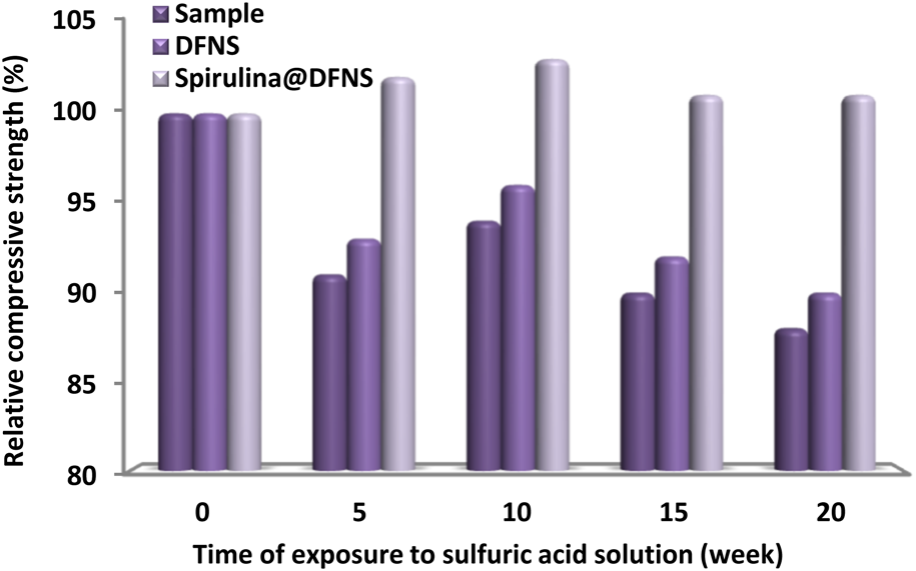
Comparison of mass variations in specimens with spirulina@DFNS and DFNS when exposed to a sulfuric acid solution.

The durability of concrete samples subjected to H_2_SO_4_ was assessed at 5, 10, 15, and 20 weeks intervals. As depicted in Fig. 14, the results demonstrate the relative resistance of the specimens to the acidic environment. Notably, after three weeks of immersion, only the concrete incorporating spirulina@DFNS exhibited enhanced resistance, likely attributed to byproducts of acid–base reactions filling internal voids. By the 10 weeks mark, while the strength of most specimens declined, those containing spirulina@DFNS continued to display improved resistance. Following 20 weeks of acid exposure, all samples showed a significant decrease in compressive strength; however, the reduction was less severe in concrete modified with spirulina@DFNS, highlighting its enhanced performance as an additive. After 10 weeks of exposure, the concrete with DFNS showed enhanced resistance, but the specimens containing spirulina@DFNS displayed even greater durability in the acidic environment, highlighting their superior ability to protect concrete from sulfuric acid damage.

**Fig. 15:**
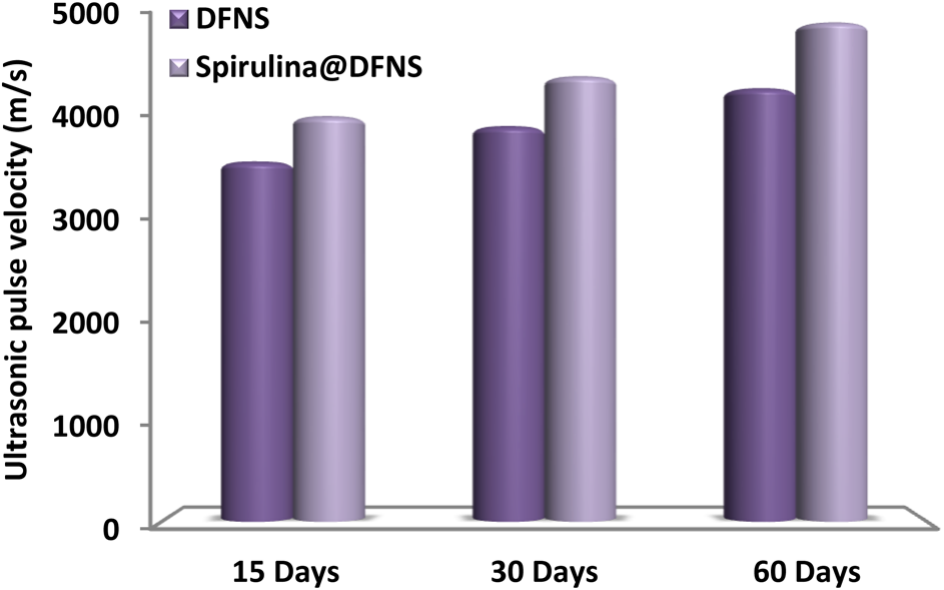
Ultrasonic test results for various ratios of nanoparticles after 15–60 days.

Fig. 15 illustrates that concrete mixtures incorporating spirulina@DFNS and DFNS exhibited greater ultrasonic wave velocities compared to the control sample. This increase implies that these additives effectively reduce voids within the concrete, facilitating faster wave propagation through the specimens. The ultrasonic test results align with the findings of the splitting tensile strength assessments, which also indicate that samples containing spirulina@DFNS achieve a higher transient pulse velocity than the others. This enhancement is due to spirulina@DFNS and DFNS filling microvoids, bonding with coarse cement particles, and promoting a denser, more compact concrete matrix.

**Fig. 16:**
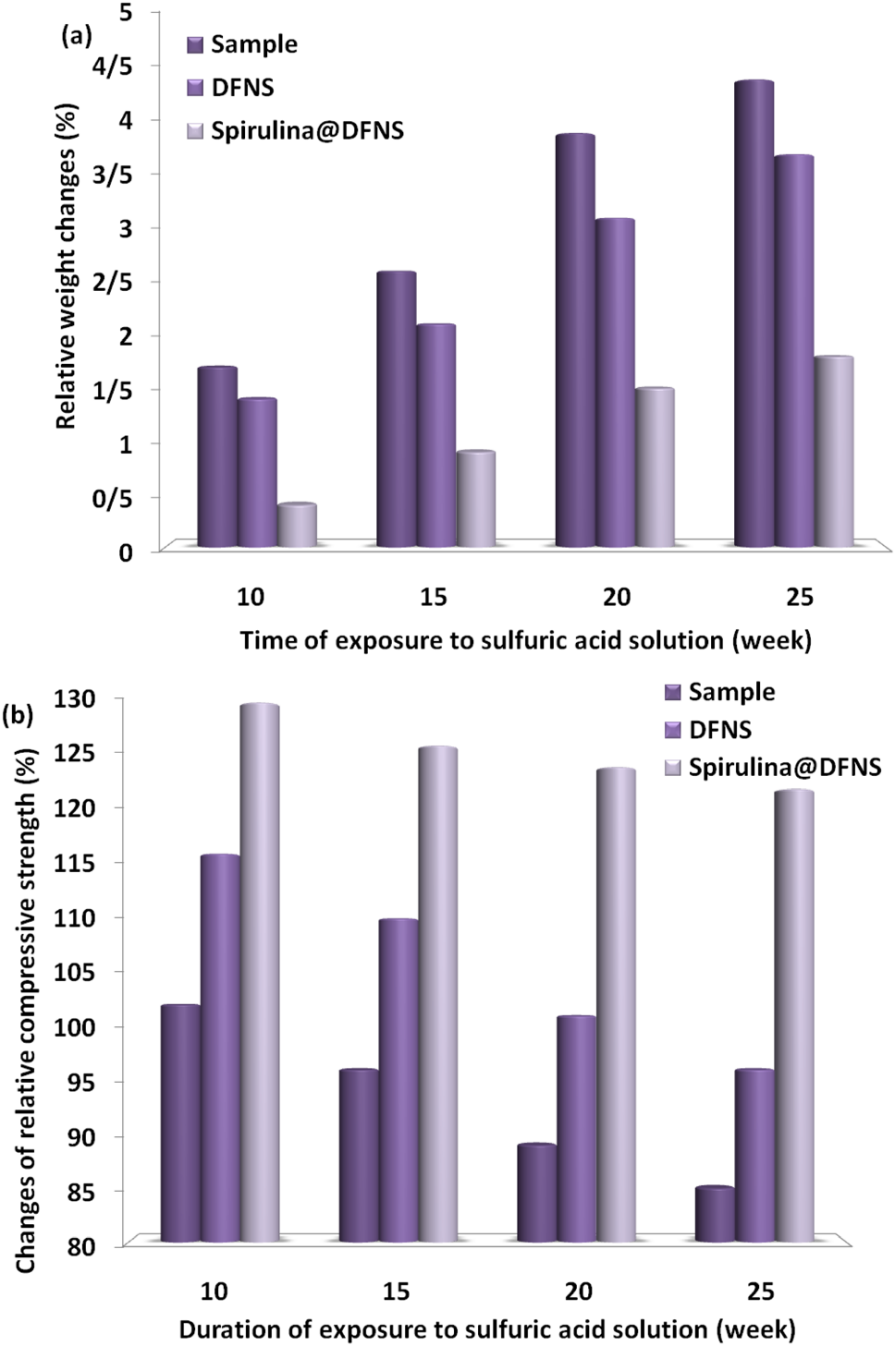
(a) Changes in relative weight and (b) variations in relative compressive strength of concrete samples containing spirulina@DFNS and DFNS in H_2_SO_4_.

Concrete samples with spirulina@DFNS showed increased resistance to sulfate damage. After 25 weeks of exposure to the acidic solution, the concrete demonstrated a weight loss of approximately 1.8%, which was the smallest loss observed among all the samples tested. In sulfuric acid (Fig. 16a), the concrete containing spirulina@DFNS exhibited lower weight loss than both the sample without nanoparticles and the DFNS-containing sample. Compressive strength tests were conducted on the concrete specimens exposed to H_2_SO_4_ at 10, 15, 20, and 25 weeks intervals. After 10 weeks of exposure, only the spirulina@DFNS specimen showed an increase in compressive strength, likely due to the formation of byproducts from acid reactions that filled the voids within the concrete. By the 15th week, the spirulina@DFNS sample exhibited a continuous rise in compressive strength, whereas the other samples showed a decline in strength (Fig. 16b). At the 25 weeks mark, the spirulina@DFNS sample exhibited superior compressive strength retention, whereas the other samples experienced a more pronounced decrease, showcasing its better resistance to acid-induced damage.

**Fig. 17:**
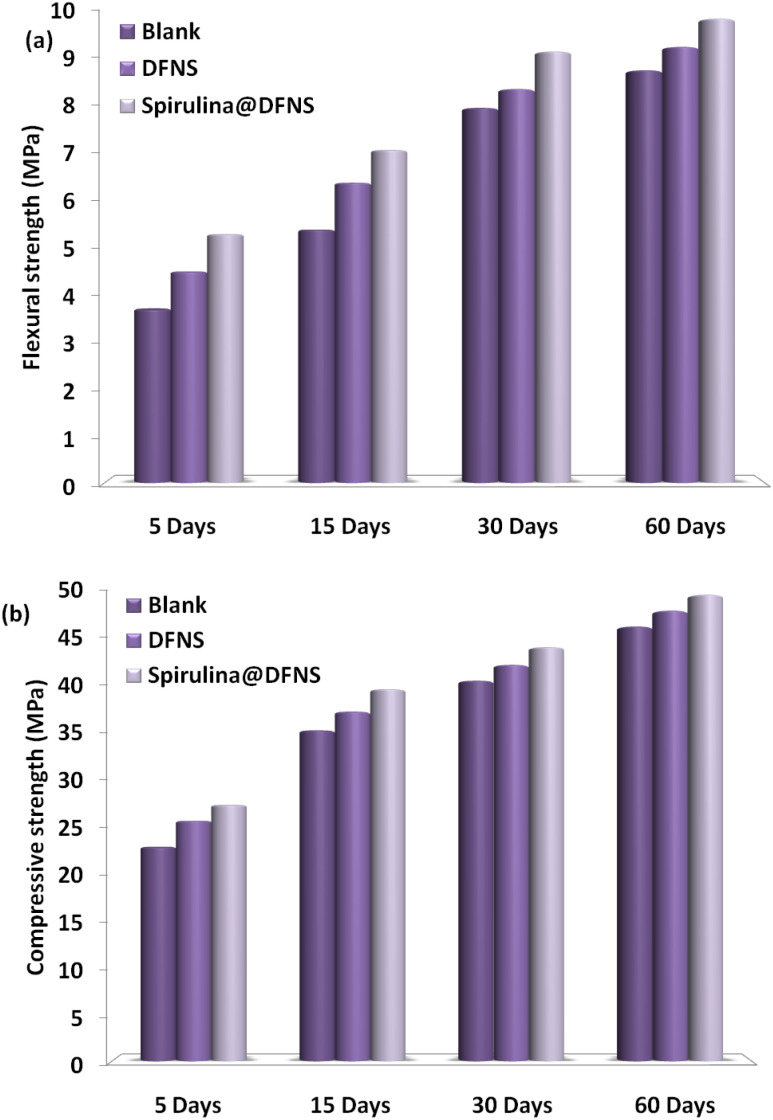
The effect of spirulina@DFNS and DFNS on (a) flexural strength and (b) compressive strength of cement mortar.

Fig. 17a presents the impact of spirulina@DFNS and DFNS on the development of compressive and flexural strengths in hardened mortar over curing periods of 5, 15, 30, and 60 days. At curing periods of 5, 15, 30, and 60 days, the cement containing DFNS exhibited flexural strength comparable to the control mortar, whereas the cement incorporating spirulina@DFNS demonstrated enhanced performance. Adding DFNS and spirulina@DFNS led to a marked increase in cement mortar compressive strength, with spirulina@DFNS delivering the stronger effect. The enhancement in compressive strength is primarily due to the high pozzolanic activity of spirulina@DFNS, which enhances cement uptake and promotes Ca(OH)_2_ hydration in the interfacial transition zone between the aggregate and cement paste. spirulina@DFNS performs better than DFNS mainly because its superior dispersibility and reduced agglomeration enhance its reactivity. spirulina's pozzolanic behavior is attributed to the existence of surface flaws and ionized hydroxyl (–OH) groups. However, as the connection rate increased, the mechanical properties weakened, suggesting that a faster grafting process may diminish the strength-enhancing capability of these additives. Fig. 17b further highlights the correlation between compressive and porosity strength, underscoring the filling effect of spirulina@DFNS and DFNS. The data reveal an inverse exponential correlation between porosity and strength, with a noticeable effect at lower porosity levels. A notable improvement in the compressive strength of cement mortar can be achieved by effectively decreasing its porosity.

**Fig. 18:**
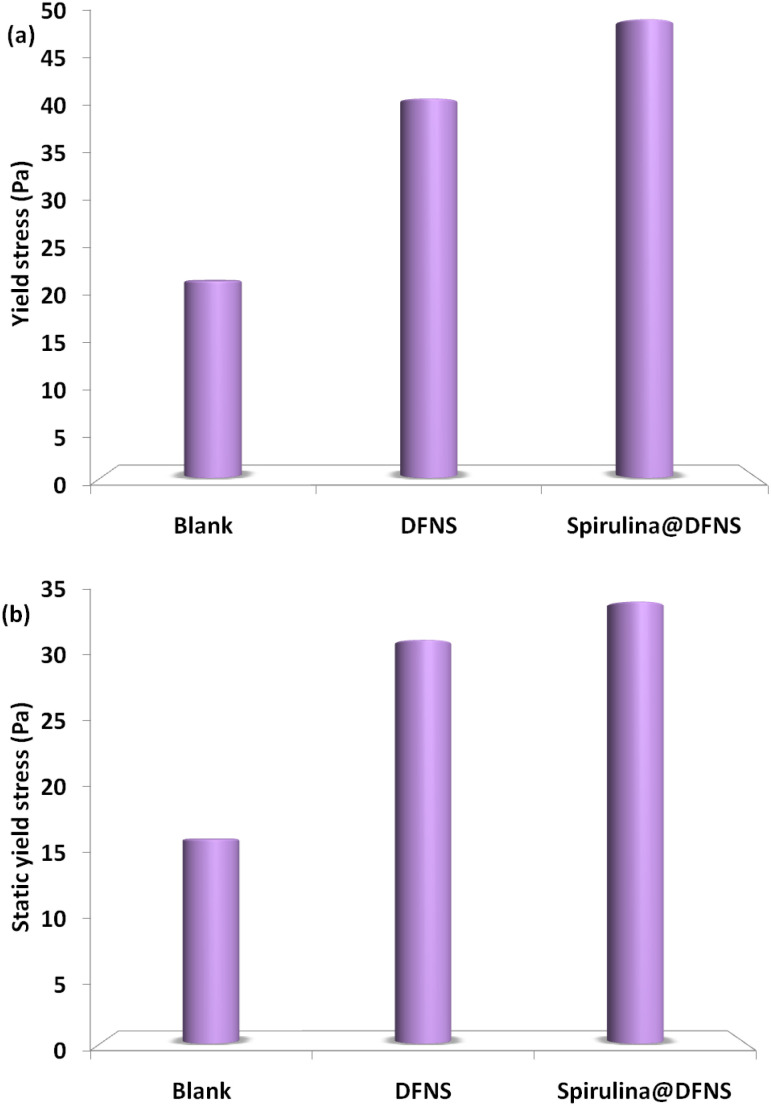
(a) Analysis of the impact of spirulina@DFNS and DFNS on the elastic limit of cement paste, and (b) depicts the influence of spirulina@DFNS and DFNS on the peak static efficiency stress.

As illustrated in Fig. 18a, the addition of spirulina@DFNS and DFNS significantly raised the elastic limit. The enhancement was attributed to the larger surface area, which promoted improved water retention in the cement mix. Greater water absorption led to a more pronounced shear thickening effect in the paste. The grafting of DFNS onto particle surfaces introduced hydrophilic properties, which became more prominent as the grafting rate increased. This process intensified water absorption, strengthening the shear force and reinforcing the influence of spirulina@DFNS. Although a higher grafting degree further amplified shear thickening, its impact on plastic viscosity remained minimal. In all cement paste samples, the elastic limit showed a consistent trend: it increased to a maximum point before leveling off. Nevertheless, the peak values differed between samples, reflecting the patterns seen in the yield stress measurements (Fig. 18b).

**Fig. 19:**
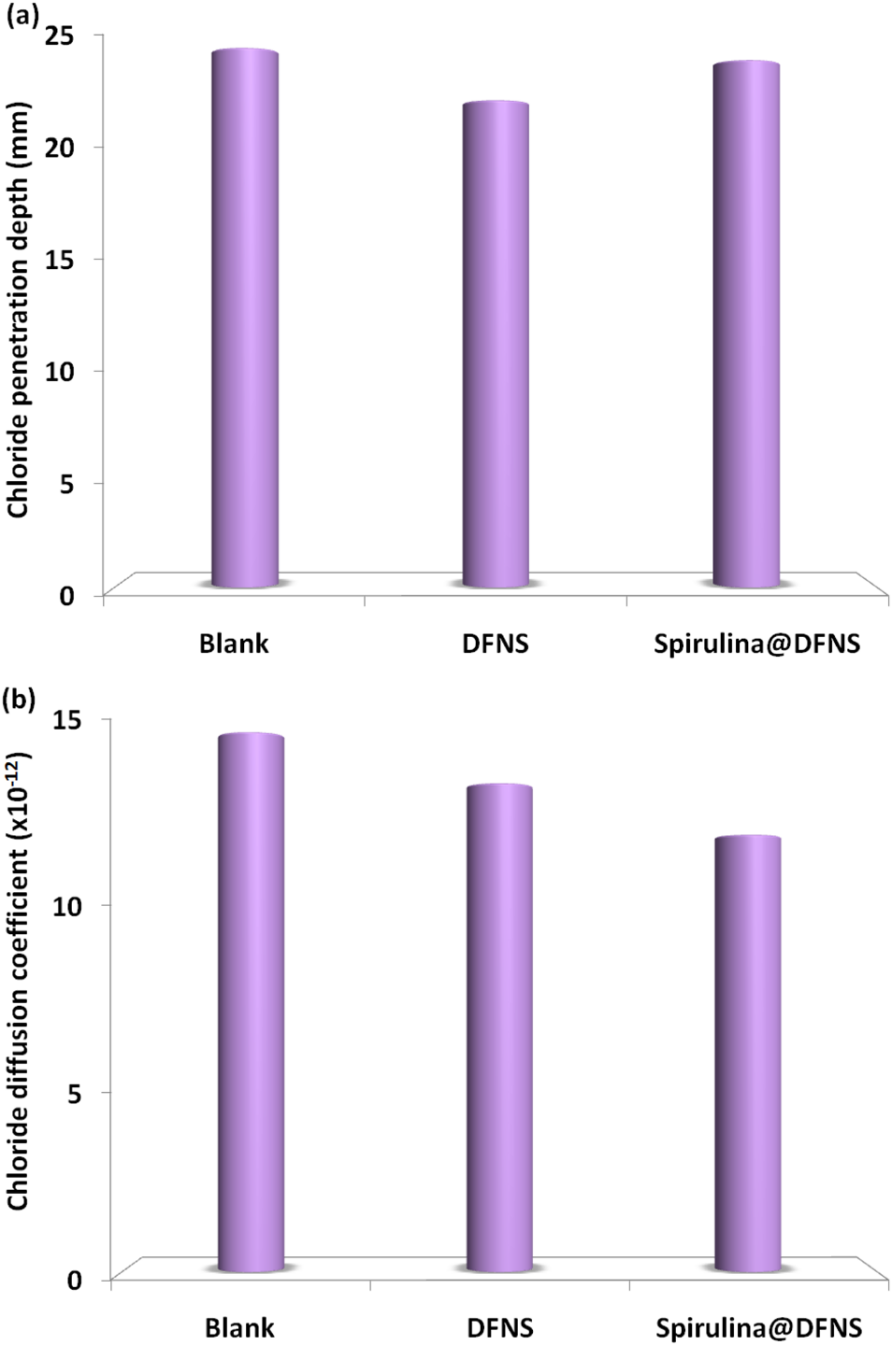
(a) Chloride penetration depth and (b) diffusion coefficient after 30 days of curing.

Previous research has shown that chloride ion-induced dissolution is the key factor affecting the durability of concrete in aquatic environments. This research investigated the durability of cement mortar samples against Cl^−^ corrosion using the rapid chloride diffusion coefficient technique. The chloride diffusion coefficient was measured following a 30 day curing period. The results (Fig. 19) showed variations in the chloride movement index and the extent of intrusion for mortar samples containing spirulina@DFNS and DFNS. Mortar samples incorporating spirulina@DFNS and DFNS exhibited greater resistance to Cl^−^ corrosion than the control samples. The spirulina@DFNS samples had the minimal chloride penetration depth and the lowest diffusion coefficient, highlighting their superior ability to withstand chloride-induced deterioration. According to ASTM C1202 classifications, the diffusion coefficient values obtained for spirulina@DFNS fall within the “low” to “very low” permeability range (<1000–2000 Coulombs), indicating a significantly enhanced resistance to chloride ion penetration. This suggests that spirulina@DFNS concrete not only outperforms the control mix, but also meets durability benchmarks relevant for long-term infrastructure exposure to aggressive environments.

**Fig. 20:**
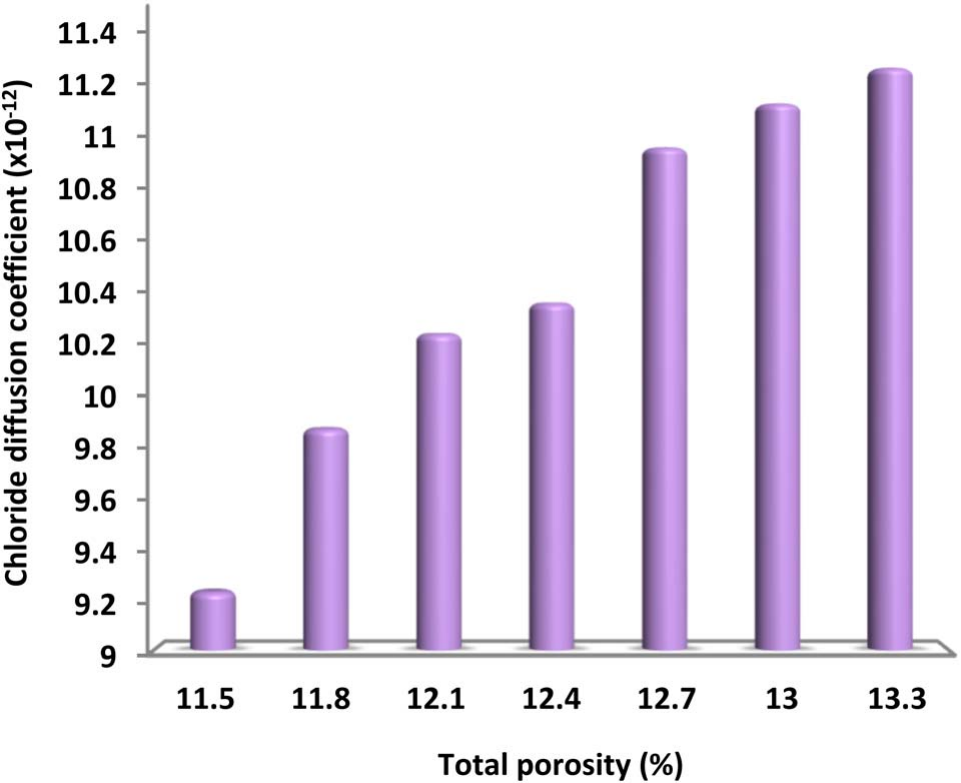
The relationship between overall permeability and the chloride diffusion coefficient at a 30 day curing age.

Fig. 20 illustrates the numerical correlation between the chloride movement index and the overall permeability. It is clear that there was a direct relationship between the Cl^−^ diffusion coefficients and total permeability. This suggests that as overall permeability increased, the chloride ion migration coefficient gradually rose as well. As the spirulina particles did not chemically react with chloride ions, the cement sample's resistance to chloride penetration can be attributed to its exceptional structural strength. This explains the link between decreased permeability and improved resistance to chloride ion-induced erosion. The chloride diffusion coefficient was considerably higher in spirulina samples compared to spirulina@DFNS. The incorporation of spirulina caused a significant alteration, greatly reducing the cement paste's liquidity and resulting in an increase in the formation of pores and defects. This assertion is supported by the MIP measurements.

To address the concern regarding the energy and sustainability implications of spirulina@DFNS incorporation in concrete, we conducted a comparative analysis between the energy input required for preparation and the performance improvements gained. Based on our process, the total energy consumption for preparing 100 grams of spirulina@DFNS is estimated at approximately 1.94 kWh, including sonication, thermal stirring, and low-temperature vacuum drying. While this represents a moderate energy investment, industrial-scale optimization can reduce this value by 30–40%. More importantly, concrete enhanced with spirulina@DFNS exhibited significant performance gains such as a 97% increase in impact energy absorption, around 20% reduction in drying shrinkage, and enhanced acid and chloride resistance leading to improved structural integrity, extended service life, and reduced maintenance requirements. When spirulina is sourced as a byproduct from wastewater treatment or uncontrolled algae blooms, the environmental impact associated with its cultivation is negligible, aligning the process with circular economy goals. In terms of cost, the lab-scale preparation is estimated at 10–15 USD per kg, but when used at dosages of 2–4% by weight, the overall cost impact per cubic meter of concrete remains under 5%, especially when offset by improved durability and reduced cement demand. These findings confirm that spirulina@DFNS concrete is not only technically viable but also energy-conscious and economically reasonable for sustainable construction applications.

## Conclusions

This research examined how partially replacing cement with spirulina@DFNS influences the mechanical and durability characteristics of concrete. The spirulina@DFNS was produced in the laboratory, with its quality validated through methods including TEM, SEM, TGA, and FTIR analysis. Due to their nanoscale size and structure, spirulina@DFNS is capable of penetrating even the finest voids and pores within the concrete matrix. By efficiently occupying these voids, they considerably improve both the splitting and compressive tensile strengths, leading to enhanced overall durability of the concrete:

- Concrete mixtures enhanced with spirulina@DFNS exhibited superior compressive and splitting tensile strengths at all curing stages compared to the control sample. Additionally, those incorporating spirulina@DFNS outperformed mixtures containing only DFNS, highlighting its greater effectiveness in strengthening the material.

- Incorporating spirulina@DFNS results in a more gradual slope in the capillary absorption curve compared to the control sample. This reduction indicates decreased water uptake, likely due to fewer and smaller capillary pores within the concrete. Similarly, the presence of DFNS also contributed to lower water absorption than that observed in the control mixture.

- Ultrasonic testing indicated an increase in sound velocity through the specimens over time. This finding aligns with the compressive and splitting tensile strength test results, confirming that specimens with spirulina@DFNS achieved greater transient pulse velocity than the other samples.

- Mortars with spirulina@DFNS showed greater compressive strength than the reference sample. spirulina@DFNS demonstrated a greater ability to disperse uniformly within the cement matrix.

- While spirulina@DFNS showed superior performance in terms of mechanical strength, durability, and dispersion, certain limitations must be acknowledged. The cost and energy associated with spirulina functionalization may affect scalability, particularly if large quantities are required. Moreover, long-term durability under field conditions and environmental aging effects require further investigation to validate the material's practical applicability.

- This evidence points to spirulina@DFNS being a viable and effective additive for cement. The pegylation technique used in this study could be readily applied to other additives, offering benefits such as lower mixing energy consumption, reduced PCE demand, enhanced viscosity, and increased compressive strength.

## List of abbreviations

DFNS: Dendritic fibro nano silica
TGA: Thermal gravimetric analysis
TEM: Transmission electron microscopy
BET: Brunauer–Emmett–Teller
SEM: Scanning electron microscope
FTIR: Fourier transform infrared spectroscopy

## Conflicts of interest

There are no conflicts to declaare.

## Data Availability

The data supporting the findings of this study are available within the article.
